# Encapsulation of Tea Polyphenol in Zein through Complex Coacervation Technique to Control the Release of the Phenolic Compound from Gelatin–Zein Composite Film

**DOI:** 10.3390/polym15132882

**Published:** 2023-06-29

**Authors:** Shabbir Ahammed, Md Easdani, Fei Liu, Fang Zhong

**Affiliations:** 1 Key Laboratory of Synthetic and Biological Colloids, Ministry of Education, Jiangnan University, Wuxi 214122, China; 2 Science Center for Future Foods, Jiangnan University, Wuxi 214122, China; 3 School of Food Science and Technology, Jiangnan University, Wuxi 214122, China; 4 Jiaxing Institute of Future Food, Jiaxing 314050, China

**Keywords:** coacervation, microencapsulation, zein, gelatin, controlled release, crosslinking

## Abstract

Green tea polyphenol (TP) was encapsulated in zein and fabricated into a gelatin–zein composite film by complex coacervation. Transglutaminase (TG) crosslinking was employed to obtain a compact structural orientation of the film to prolong the release of bioactive compounds. The encapsulation efficiency of zein and the TP release rate from the composite film were investigated. The retention rate was over 30% and 80% after film fabrication and storage, respectively. Crosslinking decreased the diffusion coefficient by half, thus improving the release of TP from the film. The antioxidant properties were satisfactory after discharge from the film detected by DPPH/ABTS scavenging. The value of crosslinking degree (~60%) and increased molecular weight of the protein were investigated by SDS-PAGE, indicating the compatibility of TP and TG treatment. According to physicomechanical findings, the TG2TP1 film exhibited the best characteristics. Tensile strength and water solubility properties were ameliorated by the TG treatment of TP-encapsulated films compared to the control film. TG and TP-loaded gelatin–zein composite film had better thermal stability than the control film. Moreover, the TP loading reduced the transparency value and improved the light-barrier properties of the film. The films showed significant antimicrobial activities against two food-borne bacteria, including *Staphylococcus aureus* BCTC13962 and *Escherichia coli* BCRC10675. The result obtained shows that the encapsulation of TP and TG treatment may be used to fabricate gelatin–zein composite film with controlled release of phenolic compounds for active packaging applications.

## 1. Introduction

Encapsulation is the process of entrapping an active compound with wall materials to shield a delicate object in the form of micro- and/or nanostructures. Different encapsulation technologies, such as electrospraying, electrostatic spray drying, and so on, have been developed for industrial applications but they have some drawbacks. Multinucleated capsules are created during the drying process and, since the essential oil globules are scattered both within and on the particle’s surface, volatiles may be lost as a result [1]. Moreover, spray drying requires expensive equipment, which increases the cost of production. The alternative technique of spray drying for encapsulation could be the liquid–liquid dispersion method. Phenolic compounds can be encapsulated by coacervation, which involves encouraging electrostatic interactions between positively and negatively charged polymers, resulting in the separation and creation of complex, neutral-charged polymers [2].

Zein, obtained from corn endosperm, is positively charged in aqueous ethanol solutions. Zein has a special solubility that makes it possible to quickly lower the alcohol concentration by adding it to the water to solidify zein droplets as micro- or nanosized particles, a process defines as liquid–liquid dispersion [3]. Gelatin type B is negatively charged over pH 5 [4]. Therefore, the positively charged zein molecule loaded with the active compound can form microencapsulation by adding a positively charged gelatin solution due to the sudden reduction of alcohol concentration.

Active components made of natural functional elements encapsulated in biodegradable polymer could create packaging with functions to alter plastic packaging in the food industry and reduce tremendous environmental pollution [5]. Oxidation can result in low molecular weight and flavorless compounds, lowering the dietary value, the shelf life of the food, and the consumer-friendly quality of food [6]. The storage of food and the maintenance of freshness depends heavily on antioxidant packaging [7]. Black or green-tea polyphenol (TP) has several beneficial qualities, including oxidation resistance, anticancer capabilities, and antibacterial properties [8]. Many researchers have reported that the incorporation of TP enhanced the antimicrobial property of gelatin [9], starch [10], and pectin–konjac glucomannan composite film [11]. TP has fewer adverse effects than artificial additives and is more effective at preventing the buildup of several toxins in the body. Recently, scientists added TP directly to films to increase their antioxidant properties [12] but the food is not safe as soon as they are consumed in the reaction and loses quality quickly [13]. Therefore, the phenolic compound needs to be encapsulated to protect and control the release behavior, as those are highly volatile. Moreover, the existing films showed either poor mechanical properties or water solubility which need to be improved for packaging application.

Controlled release of active compounds and satisfactory physical properties are two aspects of active packaging, a new packaging method intended to keep food safer and healthier. Crosslinking increases polymerization and enhances the compactness of the film [14]. Transglutaminase (TG) has been reported to crosslink between homogeneous and heterogeneous protein molecules where the optimal pH (5–8) was the precondition for enzymatic activity [15]. In addition, crosslinking has been reported to delay the release of phenolic compounds [16] as well as the mechanical and water resistance property [15].

This research was carried out by mixing TP with zein, and the TP-loaded zein microparticles were formed by the liquid–liquid dispersion method following the addition in a gelatin solution. TG crosslinking was carried out to form a compact film structure to prolong the release of TP from the film. To verify the encapsulation efficiency of TP in zein was examined, the release behavior was checked. After that, the antioxidation properties of the film were evaluated by DPPH/ABTS^+^ scavenging method. The crosslinking degree and the changes in the polymer’s molecular weight were evaluated in SDS-PAGE. The films were characterized by checking mechanical properties and some qualitative analyses following the ASTM standard method. The thermal and structural characterization was carried out by DSC, SEM, and FTIR. Finally, the film was tested for antimicrobial activity.

## 2. Materials and Methods

### 2.1. Materials

Gelatin (bloom ≥ 250 g) was commercially obtained from Rousselot (DA’AN) Gelatin Co., Ltd., (Jilin, China). Zein was purchased from Chuangsai Tech. Co., Ltd., (Shanghai, China). Tea polyphenol (purity ≥ 98.0) and transglutaminase (E.C. 2.3.2.13, activity 1364.1 U/g) were parched from Chengdu Wagott Bio-Tech Co., Ltd. (Chengdu China) and Jiangsu Yiming Bio. Co., Ltd. (Shanghai, China), respectively. Other chemicals were purchased from Sinopharma at laboratory grade and used in the experiment without additional purification.

### 2.2. Preparation of TP-Loaded Zein Microparticles and Film

Zein at 4 mg/mL was dissolved in an 85% (*v*/*v*) ethanol aqueous solution. After 2 h of continuous stirring at room temperature, tea polyphenol at 0.8 mg/mL or 1.6 mg/mL was added and continued stirring for 30 min. The zein–tea polyphenol mixture was added drop by drop into the gelatin solution of 80 mg/mL in water. After that, a transglutaminase (1 or 2 wt%, on a protein dry basis) was added and heated at 45 °C for 30 min. Finally, the film-forming solution (FFS) was heated at 80 °C for 1 min to deactivate the enzyme; 10 g of the solution was cast on a 100 cm^2^ square Petri dish. TP or TG did not add to the film-forming solution in the control film.

### 2.3. Retention of TP

Quantifying the retention of TP after film fabrication and 14 days of storage was done using modified methods [17]. The film specimen of 1 cm^2^ was kept in an ambient desiccator with a relative humidity of 53%. The specimen was taken in water (10 mL) and kept under stirring for 8 h at 40 °C. By the Ferrous tartrate method, 1 mL supernatant was taken for a UV test at 540 nm after centrifuging the mixture at 5000× *g* for 30 min. The retention of TP was computed using Equations (1) and (2):

(1) $$Retention\;after\;film\;formation\;(\%)=\frac{M1_{d}}{M0}\times 100$$

(2) $$Retention\;during\;storage\;(\%)=\frac{M14_{d}}{M1_{d}}\times 100$$

where *M*0 stands for the initial amount of TP in the FFSs, *M*1*_d_* for the first day of the dried film, and *M*14*_d_* for the fourteenth day at storage.

### 2.4. One-Way Release Measurement

The release of encapsulated TP from the film was measured following the procedure of Kuai [18] with minor modifications. Briefly, a glass bottle (volume 50 mL) with PTFE/silicon septum with a screw cap was poured with 25 mL of deionized water. The film sample was cut into spherical shapes to fit the exterior diameter of the bottle. The entire bottle was wrapped in aluminum foil after the cap was screwed on firmly to ensure a strong seal. The bottle was turned upside down so that the specimen could come in contact with water and begin the one-way release of TP. The experiment was conducted at 25 ± 1 °C. The sample (0.5 mL) was collected for the UV test. The TP concentration in the film was measured as Section 2.3. Every measurement was made in triplicate, and the release kinetics were computed using Equation (3):

(3) $$\frac{M_{t}}{M_{p}}=\frac{4}{L_{p}}{\left(\frac{Dt}{\pi }\right)}^{1/2}$$

where *M*_P_ represented the initial TP loaded in the film, *L_p_* represented the thickness of the film, and *D* represented the diffusion coefficient. *M_t_* represented the mass of TP in the deionized water at a specific time *t* (s). *M_t_*/*M_p_* vs. *t*^1/2^ was plotted and *D* was calculated from the slope.

### 2.5. Antioxidant Activity

The antioxidant activity of the freshly prepared film was determined by the procedure of [19]. The film specimen was added with water at a 0.05 gm/mL concentration and heated at 37 °C for 8 h with gentle shaking. Active DPPH methanol solution or ABTS solution (2 mL) was mixed with the supernatant (2.5 mL) and kept in the dark for 30 min.

A UV spectrophotometer was used to record the absorbance at 517 nm and 734 nm for the DPPH and ABTS^+^ scavenging activity, accordingly. The DPPH or ABTS^+^ scavenging activity (R (%)) was calculated by Equation (4):

(4) $$R\left(\%\right)=\left(1-\frac{A_{s}-A_{b}}{A_{c}}\right)\times 100\%$$

where *A_b_* is the absorbance of the sample diluted with water or PBS, *A_s_* is the absorbance of the sample, and *A_c_* is the initial absorbance of DPPH or ABTS^+^.

### 2.6. Crosslinking Degree and SDS-PAGE

The method outlined [20] with minor modification was employed to determine the crosslinking degree. NaHCO_3_ of 0.1 M was used to dissolve the film and TNBS at 1 mg/mL and 0.01%, respectively. The mixture of film and TNBS solution (film: TNBS 2:1 mL/mL) was heated at 37 °C for 2 h. Then, 5 mL of film solution and 2.5 mL of TNBS solution were mixed and heated at 37 °C for two hours. After that, 1.25 mL HCl (1 N) and 2.5 mL SDS (10%, *w*/*v*) were added to the mixture. An identical procedure was used to prepare the blank, but no films were used. The absorbance was recorded by spectrophotometer at 345 nm in comparison to the blank, and the crosslinking degree was measured as Equation (5):

(5) $$Crosslinking\;degree\left(\%\right)=\left(1-\frac{A_{c}}{A_{0}}\right)\times 100$$

where *A_c_* and *A_0_* are the absorbances of crosslinked and control film, accordingly.

The changes in molecular weight after transglutaminase crosslinking were detected by SDS-PAGE. The sample was prepared by dissolving the film in 1 mg/mL in 0.5 mg/mL SDS solution. Prior to centrifuge at 10,000× g, the sample mixture was heated at 95 °C for 30 min. Stain-Free kit, 12% from Bio-Rad Laboratories, Inc., California, USA, was used to prepare the gel. A protein marker of 10–250 kDa from the same company was used as the standard.

### 2.7. Physical, Structural, and Mechanical Characterization

The strength and flexibility of the films were determined by following the method. Briefly, zein film strips (1 × 5 cm) were placed in a texture analyzer (model no. TA XT2i, Lloyd instruments, Surrey, UK) and force was applied following the ASTM D882 standard testing method until the film was broken. The thickness was recorded randomly at seven points by a micrometer. The test was run five times with the average result being used.

Film strips (1 × 5 cm) were weighed (±0.0001 g) to determine the initial weight. The film strips were heated continuously by using an oven at a temperature of 105 °C to obtain a constant weight (W_0_). The moisture percentage was calculated using lost weight. The dried film samples were immersed into 30 mL of deionized water and stirred constantly at 50 rpm for 24 h at 35 ± 1 °C. The undissolved portion of the film was separated by filtration and dried at 105 °C, as described above, to determine the weight (W_1_) of the insoluble contents. The solubility of the film in water was calculated by Equation (6):

(6) $$Water\;solubility\left(\%\right)=\frac{W_{0}-W_{1}}{W_{0}}\times 100\%$$

In attenuated total reflection (ATR) mode, FTIR spectra of the zein films were obtained (model no. Nicolet IS 50, Thermo Electron, Waltham, MA, USA). The wavenumbers ranged from 650 to 4000 cm^−1^, and 32 scans were taken at 4 cm^−1^ resolution.

A differential scanning calorimeter (model no. DSC 8500, Perkin Elmer, Waltham, Massachusetts, USA) was used to measure the denaturation temperatures of the zein films; 3–5 mg of the film was taken into an aluminum pan, the lid put on it, and sealed. For two minutes, the temperature was held steady at 20 °C while being heated at 10 °C/min from 20 to 220 °C. After that, the sample was cooled to 0 °C and heated again from 0 to 160 °C at the same rate. The same aluminum pan without a sample was used as a reference.

The film strips were broken, immerging into liquid nitrogen to obtain the cross section. After coating with gold, the morphology was recorded using an SEM (SU8010, Hitachi, Japan).

### 2.8. Color and Transparency

The color of the gelatin–zein film was determined by the method of [21] using a colorimeter (model UltraScan Pro1166, Hunter associates laboratory, Inc., Reston, Virginia, USA) spectrophotometer. The parameters measured compare with a white standard (L* = 93.64, a* = −0.96, and b* = −0.45) where L* = lightness, (a*) =redness and b* = yellowness. Finally, the index of total color difference (ΔE*) was determined as Equation (7):

(7) $$∆E*=\sqrt{{{\left(∆L*\right)}^{2}{+\left(∆a*\right)}^{2}+\left(∆b*\right)}^{2}}$$

The ΔL*, Δa*, and Δb* were the difference between the obtained value and the white standard.

The transparency value of the film was measured by using a (model TU-1810, Purkinje Ins. Ltd., Beijing, China). Light of 200–800 nm wavelengths was transmitted through the film to calculate the transparency.

(8) $$transparency\;value=\frac{-\log T_{600}}{x}$$

where *T*_600_ = the fractional transmittance at wavelength 600 nm and *x* = film thickness in nm.

### 2.9. Antimicrobial Properties

*Staphylococcus aureus* BCTC13962 and *Escherichia coli* BCRC10675 were obtained from the laboratory of the brewing (Wuxi, China). First, the test strain was aseptically inoculated into Tryptone Soy Broth (TSB; Solarbio Sci. & Tec. Co., Ltd., Beijing, China) and incubated overnight at 37 °C. The concentrations of the *S. aureus* and *E. coli* suspensions were ≈1 × 10^9^ CFU/mL and ≈5 × 10^9^ CFU/mL, respectively. To reach an initial concentration between 10^7^ and 10^8^ CFU/mL, 200 µL of bacterial culture was aseptically taken to 4.8 mL of TSB broth containing 2.5 mg of the film sample. The mixture was then incubated at 37 °C for 3 h with shaking at 100 rpm. To plate with Tryptone Soy Agar (TSA) for *S. aureus* and Luria–Bertani (LB; Qingdao Hope Bio-Technology Co., Ltd., Shandong, China) agar for *E. coli*, 0.1 mL of the mixture was sampled. Colonies were counted after the plates had been cultured for 1.5 days at 37 °C. As a control, bacterial suspension (0.5 mL) devoid of film sample was employed. The percentage of inhibition was determined by Equation (9):

(9) $$Inhibition\left(\%\right)=\frac{CFU/{mL}_{control}-CFU/{mL}_{sample}}{CFU/{mL}_{control}}\times 100$$

### 2.10. Statistical Analysis

The data were statically analyzed by using a one-way analysis of variance (ANOVA). Duncan’s multiple range test was used to determine the significant differences between mean values (*p* < 0.05). The analysis was conducted using SPSS (SPSS 16.0, SPSS Inc., Chicago, IL, USA) software.

## 3. Results and Discussions

The primary goal of this investigation was to evaluate the effectiveness of encapsulating tea polyphenol (TP) in zein by a coacervation technique and characterization of the biodegradable active composite film. Initially, the encapsulation capacity and release of TP were investigated. Furthermore, the structural and thermal characteristics of the film were investigated to ascertain its applicability.

### 3.1. Retentivity and Release of TP from the Film

**Figure 1:**
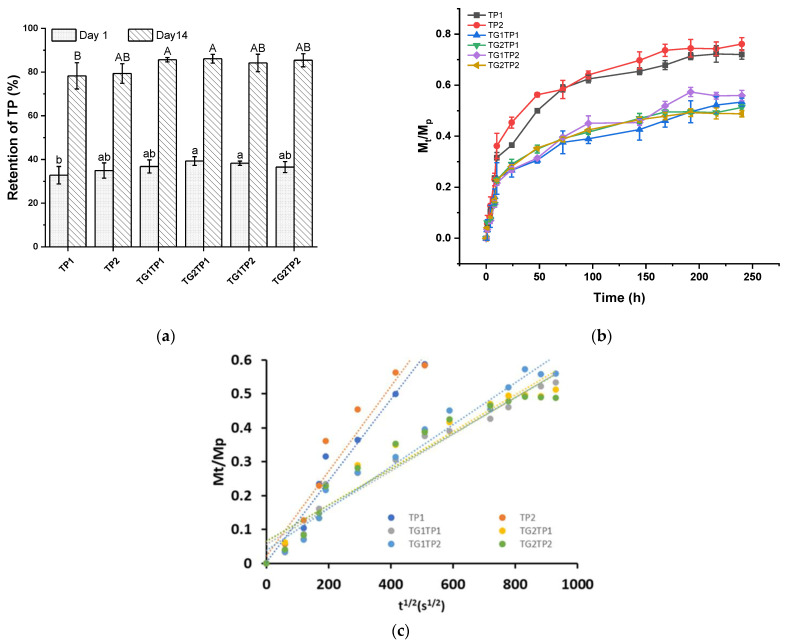
The retention of tea polyphenol in the gelatin–zein composite film (**a**), release of TP from films in water as a function of time (**b**), and *M*_t_/*M*_p_ vs. t^1/2^ (**c**). TPx and TGx: films prepared with tea polyphenol (TP) and transglutaminase (TG) at the indicated percentage of protein *w*/*w* in the composite film-forming solutions. Values are given as mean ± SD (*n* = 3). Different subscript letters in the same column indicate significant differences (*p* < 0.05).

The retention rates of TP after drying the film and storage after two weeks were determined (Figure 1a). The retention rate of TP1 and TP2 was 32.80% and 34.92% after encapsulation, accordingly. The retention was reported at 49.73% in the gelatin film, ~37% in the zein film, and 24.46% in the gelatin–zein composite film where TP was added without encapsulation [17]. The TP entrapped into zein and formed water-insoluble particles were probably responsible for the lower retention rate [18]. The crosslinking improved the TP retention rate slightly compared to films without crosslinking [22]. TG crosslinking (TG1TP1, TG2TP1, TG1TP2, and TG2TP2) increased the retention rate to 36–39%. The TP remained as a free state in the crosslinked film which resulted in an increase of the value. After two weeks of storage, the retention rate of TP was over 78% and 84% without and with the TG-treated film, respectively.

The release profile of TP from the composite films was tested by exposing the film to deionized water (Figure 1b). In general, elements that control the release of TP from the films include the degree of hydration and swelling matrix, interaction mechanism of polymer and phenolic compounds, and type of simulant used [23]. The composite films displayed comparable release characteristics, with a fast initial release due to short-term protection and a continuous gradual release. During the encapsulation process, a part of unencapsulated TP remained in the film matrix at a free state responsible for initial fast release. TP2 showed the highest release of phenolic compounds over time (Figure 1b). TG crosslinking could increase the encapsulation efficiency of TP into the film, which decreased the TP release from the film. After TG treatment, the release of TP from the film decreased by one third compared to the film without TG treatment (TP1, TP2). In addition, the crosslinking prevented the penetration of water in the film matrix which delayed the release of TP. The release of TP could be controlled by encapsulation and crosslinking for 240 h.

As shown in Figure 1c, the model successfully matched the experimental data (R^2^ > 0.9), and D values are depicted in Table 1. The encapsulation of TP controls the release process reported previously [24]. Additional treatment such as crosslinking improves the release of kinetic furthermore. The samples without TG had a double-D value than the film without TG. The crosslinking by TG formed a more robust network which prevented plasticization and a decrease in D values after immersing in water. TG crosslinking promoted the slower release of encapsulated and free-state TP from the film. The D values decreased from 1.2 × 10^−11^ to 0.5 × 10^−11^ cm^2^/s. TG crosslinking increases the compactness and tortuosity of the TP diffusion path, which prolong the release of TP [25]. In similar-type films, the release of TP was faster than the films containing a higher TP percentage (TP2 > TP1). Overall, TG-treated films with a prolonged released profile.

### 3.2. Antioxidant Activity

**Figure 2:**
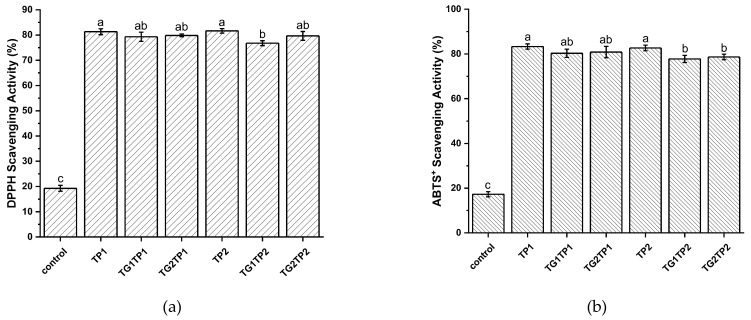
Antioxidant activity by DPPH*-SA assay (**a**); and antioxidant capacity by ABTS*-SE assay (**b**) of TP encapsulated and TG crosslinked gelatin–zein composite films. Control film prepared without TP or TG. TPx and TGx: films prepared with tea polyphenol (TP) and transglutaminase (TG) at the indicated percentage of protein *w*/*w* in the composite film-forming solutions, accordingly. Values are given as mean ± SD (*n* = 5). Different subscript letters in the same column indicate significant differences (*p* < 0.05).

The antioxidant activity of the TP encapsulated, and TG crosslinked films, was assessed using the DPPH and ABTS free-radical scavenging assays, and the results are shown in Figure 2. The control film showed some antioxidant activity, which could be assigned to the antioxidant properties of the Latin peptide [26]. Overall, the TP-loaded films showed DPPH and ABTS+ radical scavenging of 76–81% and 77–83%, accordingly. The values were highest after TP-loading in the film without crosslinking. The various concentrations of TP did not significantly differentiate antioxidant activity from one another (TP1, TP2). The result confirms the activity of phenolic compounds after encapsulation and film fabrication. However, the crosslinking increased the compactness of the films; thus, TP was entrapped in the film matrix. In TGTP-treated films, the values were around 79%, confirming the activity of TP in the crosslinked films, although the antioxidant activity decreased noticeably (*p* > 0.05) after crosslinking. In TG1TP2, the TP percentage (2%) was higher than TG (1%) in this film, which reduced the crosslinking degree to 54.95% compared with TG1TP1 (60.46%) (Table 1) and influenced the enzyme activity. Antioxidant activity increased again while 2% of both TG and TP was used (TG2TP2).

### 3.3. Crosslinking Degree

Gelatin–zein composite films treated with TG at 1% and 2% (*w*/*w*) of the protein concentrations were examined for their degree of crosslinking with or without TP (Table 1). A reduction in amino acid groups in FFS treated with TG (1%) resulted in a crosslinking of 54–60%. The accessible amino groups were further reduced when the enzyme concentration was increased to 2% (TG2), and the degree of crosslinking was increased to 59–62%. The result confirms the crosslinking but differs significantly with the concentration of TP and TG. The crosslinking degree was 60% when utilizing 1% TG (TG1TP1) in the presence of 1% (*w*/*w*) TP in the FFS, and it increased to 62% when using 2% TG (TG2TP1). Similarly, the crosslinking increased with TG percentage from 55% to 59% in the presence of 2% (*w*/*w*) TP with an increase of the TG percentage in the FFS. The increase of crosslinking degree with increasing TG concentration in gelatin was reported [27]. Interestingly, the crosslinking degree remained the same (60%) when the TG and TP ratio was the same (TG1TP1, TG2TP2) in the FFSs. However, the crosslinking degree decreased significantly when the TP percentage (2%) was higher than TG (1%) in the FFS (TG1TP2). There was no significant difference in crosslinking when the TG percentage was the same or higher than the TP percentage in the FFS, suggesting that the enzyme concentration should be the same or higher than the TP concentration for optimum crosslinking. The unencapsulated TP that remained as a free state in the FFS was responsible for the different crosslinking percentage.

**Figure 3:**
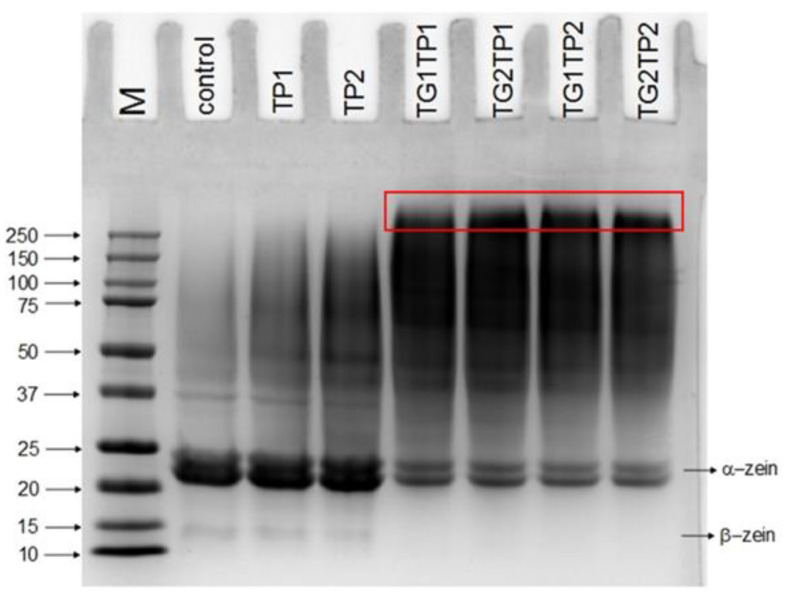
SDS-PAGE of control, without or with TG-crosslinked TP-encapsulated films. M indicated the protein marker. The red box indicates the molecules over 250 kDa.

In order to observe the changes at the molecular level, SDS-PAGE was carried out (Figure 3). The control and untreated films (TP1, TP2) showed a light band at 14 kDa and a deep band at 22–24 kDa of β- and α-zein, accordingly. Gelatin showed bands through the whole lane due to its heterogeneous molecular weight. In TG crosslinked films, the intensity of α-zein decreased and molecules with over 250 kDa were observed. The polymerization by transglutaminase was responsible for increasing the molecular weight. The result was consistent with our previous study [15].

### 3.4. Physical Properties of the Films

The tensile stress (TS) and elongation at break (EAB) of the films were shown in Table 1. TS of the films decreased after TP loading (TP1 and TP2) compared to the control film (108 MPa). However, the TS of TP-loaded films increased to over 100 MPa after TG crosslinking. The intermolecular gap was reduced as a result of the crosslinking, making the treated film more compact/rigid, resulting in a reduction in tensile strength [28]. The film loaded with TP and treated TG showed a similar TS value with TP1, in a range between 100 and 103 MPa (Table 1). However, TG2TP2 film had a significantly lower TS value (87 MPa) than other TGTP-treated films. The higher amount of TP (2%) contributed to the free TP in the FFS and the entrapped compactness might be the reason for decreasing TS. The EAB was between 5.5% and 10.5% (Table 1). The addition of TP-encapsulated films was more flexible than TG-treated films. The crosslinking degree decreased in the presence of TP loaded over 2%. The lower crosslinking formed a weaker structural film; thus, there might be a void that improves the ductility of the film. Interestingly, TP encapsulation or TG crosslinking had no significant effect on thickness (0.06–0.07 mm). Although the thickness increased compared to the control film (0.05 mm), this was attributed to the higher amount of solid content (TP1, TP2) and the formation of higher molecular weight molecules by crosslinking [29].

**Figure 4:**
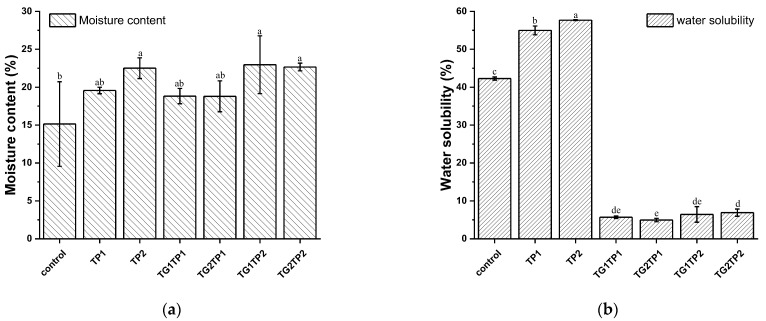
Moisture content (**a**); and water solubility (**b**) of TG crosslinked gelatin–zein composite films prepared with different percentages of tea polyphenol and transglutaminase. Values are given as mean ± SD (*n* = 3). Different subscript letters in the same column indicate significant differences (*p* < 0.05).

The moisture content (MC) and water solubility (WS) of the film were calculated as these are the critical parameters for packaging highly moist food. The MC of the film increased after TP encapsulation but decreased after being treated with TG (Figure 4a). Comparing the film to the control film (15%), TP raised the moisture percentage by about 18–22%. TP-loaded films had more void in the matrix where water was entrapped during the drying of the film. As a result, the MC increased in the film and made the film more flexible during elongation. The films loaded at the same TP concentration, TG1TP1, TG2TP1 or TG1TP2, TG2TP2 showed MC with no significant difference.

The WS of the control film was 42% due to the interaction with gelatin and zein. The solubility increased to 56–58% after TP encapsulation in the film than the control film (Figure 4b). The water stacked as a free-water state in the film matrix, observed in MC (Figure 4a), contributed to the higher solubility. The water solubility of TG1TP1, TG2TP1, TG1TP2, and TG2TP2 was in a range between 4.9% and 6.9%. The TG-modified and TP-loaded films decreased the water solubility by around six times than of the control film, which made the film suitable for moist food packing.

### 3.5. Thermal Properties

**Figure 5:**
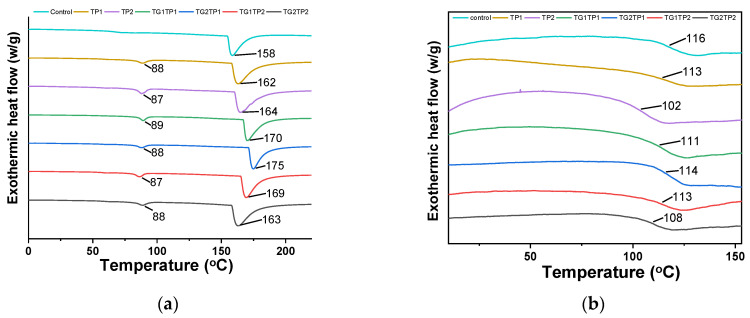
DSC thermogram of (**a**) first heating, and (**b**) second heating curve of control and TG crosslinked gelatin–zein composite films loaded with TP. Control film prepared without TP or TG. TPx and TGx: films prepared with tea polyphenol (TP) and transglutaminase (TG) at the indicated percentage of protein *w*/*w* in the composite film-forming solutions.

The use of packaging materials entails the consideration of thermal properties since packaging materials may be heated during production, heat sealing, and product consumption. DSC was used to examine the thermal behavior of the films during either heating or cooling processes. The DSC thermogram of films is shown in Figure 5a. The first peak was observed between 86 °C and 89 °C in all composite films, which represented the water evaporation [30].

The melting temperature (Tm) of control, TG-, and TP-treated films were in a range between 164 °C and 175 °C. TG2 film showed the highest melting temperature (175 °C) and the control film showed the lowest value (164 °C). TG crosslinking and TP addition at 1% (*w*/*w*) of protein decreased the melting temperature (160 °C) compared with the control film. The higher percentage (>1%) of either TG or TP increased the melting temperature of the film. TG2TP1 and TG1TP2 showed the highest melting temperature at 169 °C among combinedly treated films. However, the 2% TG and TP decreased the melting temperature TG2TP2 (163 °C). The reduction of the ordered crystal structure might be responsible for the lower melting temperature as high heat was needed to fall out of the crystal structure and melt [31]. The glass transition temperature (T*g*) was determined by the second heating scan (Figure 5b). TP-encapsulated or TG-treated films showed lower T*g* values than the control film.

### 3.6. Film’s Structure

#### 3.6.1. Secondary Structure

**Figure 6:**
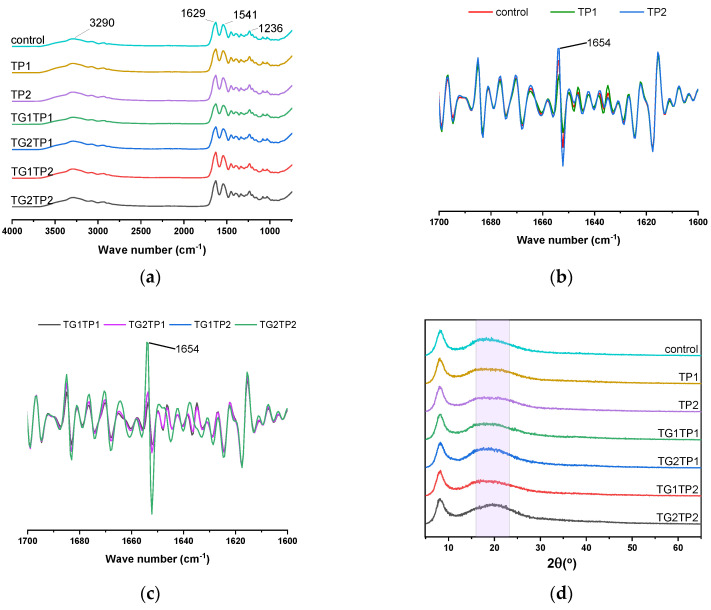
FTIR spectra of control, TP encapsulated, and combined TG and TP treated films (**a**). Second derivative FTIR spectrum of control and TP treated films (**b**); both TG and TP treated films (**c**). The XRD spectrum of gelatin–zein film (**d**).

Figure 6 represented the FTIR spectra of control, TP-encapsulated, and TG-crosslinked TP-loaded films. All films showed a distinctive peak found at 3290 cm^−1^ due to N-H stretching vibration; the peaks at 1629, 1544, and 1235 cm^−1^ represented C=O stretching, C-N stretching, and N-H bending, accordingly. To understand the changes in secondary structure, the second derivative of amide I, ranged 1700–1600 cm^−1^, was analyzed.

The presence of a single α-helix (~1654) was increased after TP encapsulation and crosslinking by TG (Figure 6b,c). TP2 and TG2TP2 showed a higher single α-helix than the control film. In order to understand the changes in secondary structure, curve deconvolution of amide I was carried out (Supplementary Figure S1). The intensity of α-helix was decreased and β-sheet was increased in the crosslinked film compared to the control film. TG crosslinking converted the α-helix to the sheet that was reported previously [32]. Among TG-crosslinked films, the higher crosslinking degree film had a lower intensity of α-helix. Collectively, the functional group was observed in all films as the control film, although the intensity was different. These findings indicated that the TP encapsulation and TG crosslinking are compatible processes for fabricating composite film.

The crystalline structure and the amorphous region of the film were evaluated by XRD. As observed, the films showed a sharp peak at 2θ about 5 degrees, indicating the crystallinity of the films had not changed significantly (Figure 6d). The strength and deformation properties of the film depend on the crystallinity [19]. The TG2TP2 films showed lower crystallinity than other TGTP films, showing a significantly lower tensile strength. The peak at 2θ about 17 degrees in the amorphous region was shifted to 19 degrees with TP loading and TG modification. The amorphous region is responsible for the flexibility of the film during deformation. The changes in the amorphous region were consistent with the elongation at break result. The transformation of α-helix to β-sheet (observed in Supplementary Figure S1) affected the amorphous region of the protein.

#### 3.6.2. Microstructure

The cross section of control and TP-loaded films were screened in Supplementary Figure S2. The TP-encapsulated films showed pronounced zein particles compared with the control film, which reduced the compactness, and an inverse effect on tensile strength was observed. The fibril structure of gelatin was lost after crosslinking, which increased the compactness of the film matrix. Chambi [33] also found that after TG modification, the loss of the fibrillar structure of gelatin was consistent with our findings. However, zein molecules formed sulfide, hydrogen, and hydrophobic bonds by crosslinking, which increased the roughness of the film [15]. The formation of larger zein particles in the film matrix prevented the development of an ordered matrix and decreased the TS. Interestingly, a smooth surface was observed in TG1TP2, which had the highest crosslinking degree, and the composite film structure was dominated by the protein network [34]. This study suggested that TG crosslinking changed the microstructure while TP had less influence on morphology.

#### 3.6.3. Optical Properties

Consumer preference is strongly influenced by the color and opacity of food packaging materials. Lightness (L*), greenness (a*), yellowness (b*), and total color difference (ΔE) of the films were evaluated and presented in Table 2. The lightness (L*) increased after being treated with TG (TG1, TG2) and decreased after adding TP (TP1, TP2) gradually compared to the untreated (control) film. In contrast, the greenness (a*) and yellowness (b*) of the film increased after adding TP, while TG did not influence these two parameters. It is of interest to note that combinedly treated by TG and TP films (TG1TP1, TG2TP1, and TG1TP2) showed the lightness (L*) value as the same as the control film. TG2TP2 contained the highest percentage of TG, and TP (2%, *w*/*w*), among all films, showed a lower lightness value than the control film. The color of TP contributed to the total color difference (ΔE). Films containing TP had a higher color difference than films without TP (control, TG1, and TG2). The polymer alignment is relevant to the transparency of the film [35]. The transparency of the film is presented in Table 2. TP-loaded films had higher transparency values and, therefore, were less transparent than control films.

### 3.7. Antibacterial Property

**Figure 7:**
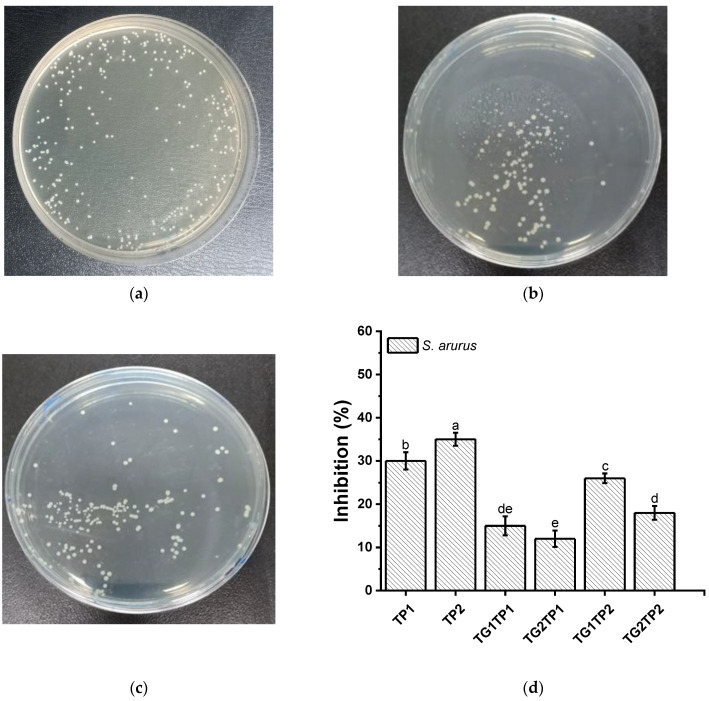

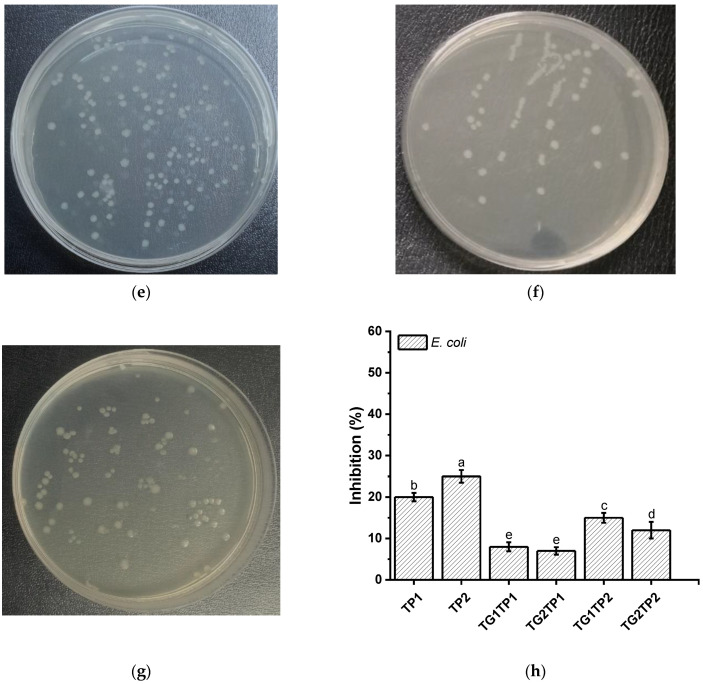
The growth inhibition (%) of *Staphylococcus aureus* by control (**a**), TP2 (**b**), and TG1TP2 (**c**). The growth inhibition of *Escherichia coli* by control (**e**), TP2 (**f**), and TG1TP2 (**g**). Graphical representation of growth inhibition by the colony-count method of all films for *Staphylococcus aureus* (**d**) and *Escherichia coli* (**h**). A 10^−4^ dilution of *S. aureus* 10^−5^ dilution of *E. coli* and were considered for the figure. Values are given as mean ± SD (*n* = 3). Different subscript letters in the same column indicate significant differences (*p* < 0.05).

TPs are polyhydroxy compounds consisting of hydroxyl groups and a hydrophobic benzene ring, which can interact with proteins of microbes via amino and carboxyl groups or hydrophobic interactions [36]. The ability to inhibit the growth of TP-encapsulated film was tested on *S. aureus* (Gram-positive) and *E. coli* (Gram-negative) bacterium (Figure 7). The TP encapsulated inhibited *S. aureus* and *E. coli* and around 30–35% and 20–25%, respectively. The inhibitory zones increased proportionally with the TP concentration in the film. Phenolic compounds can inhibit microbial growth by denaturing proteins and disrupting cell membranes [37,38]. The films with TG crosslinked showed 12–26% and 7–15% against *S. aureus* and *E. coli*, accordingly. The inhibition was lower by the TG-treated films than those without crosslinked film (TP1, TP2) due to the hydrophobic character of the films [39]. The lower diffusion rate of TP from the TG-treated film (Table 1) might be responsible for the lower inhibition percentage. TG1TP2 showed the highest inhibition percentages, which were *S. aureus* (27%) and *E. coli* (15%) among TG-treated films. The release of TP was lower from TG-treated film than from untreated film, which leads to a lower inhibition percentage.

## 4. Conclusions

Encapsulation of tea polyphenol (TP) in zein was successfully carried out by a liquid–liquid dispersion technique and fabricated a compacted structured gelatin–zein composite film incorporated with transglutaminase (TG) crosslinking. Microencapsulation improved the retention and protected the bioactive compounds. Crosslinking decreased the water solubility of the film to 10% and prolonged the release of TP from the film. The highest enzymatic crosslinking and tensile strength (102 MPa) was observed when the film-forming solution contained a higher amount of TG than TP (TG2TP1). The antioxidant and antimicrobial activity were found at a satisfactory level after TG treatment in the films. Coacervation can be used to encapsulate TP in zein and TG crosslinking enhanced the mechanical properties of the gelatin–zein active film.

## Figures and Tables

**Table 1:** Diffusion coefficient (D) of TP release, crosslinking degree of TG treated films, and mechanical properties of the films.

Sample	Release Property		Crosslinking Degree	Mechanical Property		
	D × 10^−11^	R^2^		Tensile Strength	Elongation at Break	Thickness
	(cm^2^/s)		(%)	(MPa)	(%)	(mm)
control				108.7 ± 5.9 ^a^	9.5 ± 1.9 ^ab^	0.05 ± 0.003 ^a^
TP1	1.2	0.963		97.6 ± 10.5 ^ab^	7.9 ± 1.7 ^ab^	0.07 ± 0.01 ^a^
TP2	1.2	0.931		90.9 ± 8.1 ^bc^	8.1 ± 2.3 ^ab^	0.06 ± 0.004 ^a^
TG1TP1	0.5	0.953	60.46 ± 4.96 ^ab^	100.6 ± 10.2 ^ab^	6.2 ± 0.6 ^b^	0.06 ± 0.002 ^a^
TG2TP1	0.5	0.930	62.74 ± 1.51 ^ab^	102.5 ± 4.8 ^ab^	5.5 ± 1.2 ^b^	0.06 ± 0.013 ^a^
TG1TP2	0.6	0.963	54.95 ± 6.59 ^b^	101.3 ± 7.1 ^ab^	7.5 ± 1.8 ^ab^	0.06 ± 0.009 ^a^
TG2TP2	0.5	0.922	59.05 ± 1.54 ^ab^	86.5 ± 5.3 ^bc^	10.4 ± 3.3 ^a^	0.06 ± 0.005 ^a^

Different subscript letters in the same column indicate significant differences (*p* < 0.05). Values are given as mean ± SD (*n* = 5).

**Table 2:** Color, light transmittance, and transparency values of films.

Films	Color				Light Transmittance (%)	Transparency Values
	L*	a*	b*	ΔE*	600	
control	91.86 ± 0.07 ^b^	−1.25 ± 0.01 ^g^	3.73 ± 0.25 ^f^	3.30 ± 0.05 ^g^	70.42 ± 1.64 ^c^	2.71 ± 0.18 ^ab^
TG1	92.03 ± 0.07 ^b^	−1.24 ± 0.01 ^g^	3.45 ± 0.40 ^g^	2.98 ± 0.10 ^h^	75.39 ± 0.36 ^a^	1.95 ± 0.03 ^f^
TG2	92.28 ± 0.08 ^a^	−1.26 ± 0.01 ^h^	3.31 ± 0.31 ^h^	2.76 ± 0.03 ^i^	73.11 ± 0.29 ^b^	2.03 ± 0.03 ^ef^
TP1	91.27 ± 0.06 ^c^	−0.97 ± 0.02 ^d^	4.24 ± 0.35 ^e^	4.00 ± 0.06 ^f^	69.44 ± 0.24 ^c^	2.21 ± 0.02 ^de^
TP2	90.80 ± 0.07 ^d^	−0.76 ± 0.02 ^a^	5.16 ± 0.32 ^c^	5.04 ± 0.06 ^c^	70.14 ± 0.28 ^c^	2.36 ± 0.03 ^cd^
TG1TP1	91.45 ± 0.07 ^c^	−0.99 ± 0.01 ^e^	4.43 ± 0.45 ^d^	4.09 ± 0.13 ^e^	68.65 ± 0.50 ^c^	2.63 ± 0.05 ^b^
TG2TP1	91.28 ± 0.05 ^c^	−0.87 ± 0.01 ^c^	4.45 ± 0.25 ^d^	4.19 ± 0.07 ^d^	69.13 ± 0.66 ^c^	2.51 ± 0.07 ^bc^
TG1TP2	91.03 ± 0.06 ^d^	−1.03 ± 0.02 ^f^	5.44 ± 0.29 ^b^	5.19 ± 0.03 ^b^	66.12 ± 0.78 ^d^	2.89 ± 0.08 ^a^
TG2TP2	90.67 ± 0.06 ^d^	−0.84 ± 0.01 ^b^	5.54 ± 0.31 ^a^	5.43 ± 0.03 ^a^	68.55 ± 1.10 ^c^	2.36 ± 0.10 ^cd^

L*—Lightness, a*—Redness (positive) or Greenness (negative), b*—Yellowness (positive) or Blueness (negative). Values are given as mean ± SD (*n* = 3). Different subscript letters in the same column indicate significant differences (*p* < 0.05).

## Data Availability

The data presented in this study are available on request from the corresponding author.

## Supplementary Materials

The following supporting information can be downloaded at: https://www.mdpi.com/article/10.3390/polym15132882/s1, Figure S1. Second derivative of FTIR spectrum of (a) control; (b) TP2; (c) TG2TP1; and (d) TG2TP2 films. Figure S2. SEM images of cross-section of (a) control; (b) TP1; (c) TG1TP1; (d) TG2TP1; (e) TG1TP2; and (f) TG2TP2 films.
