# Supplementary material for: Encapsulation of Tea Polyphenol in Zein through Complex Coacervation Technique to Control the Release of the Phenolic Compound from Gelatin–Zein Composite Film
Source: Polymers (Basel). 2023 Jun 29;15(13):2882. doi: 10.3390/polym15132882 (PMC10346822; doi:10.3390/polym15132882)
Supplement: Supplementary file 1 [file polymers-15-02882-s001.zip › polymers-2459700-supplementary.pdf]

Supplementary Data

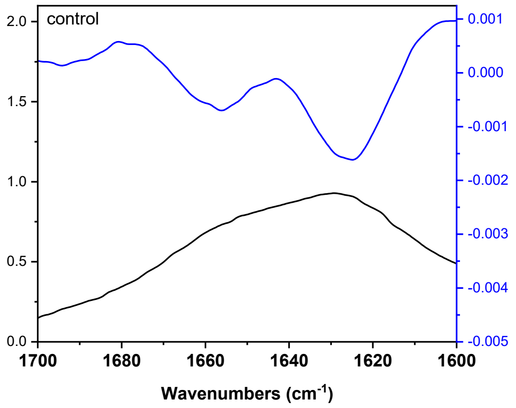

(a)

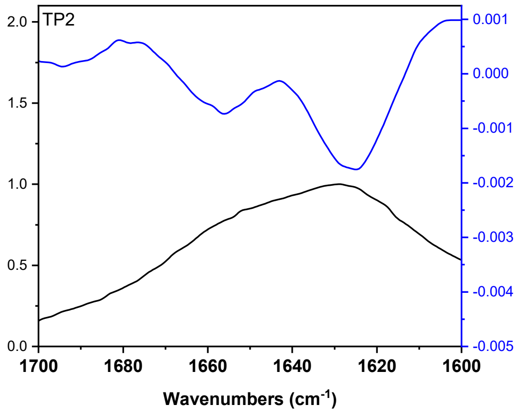

(b)

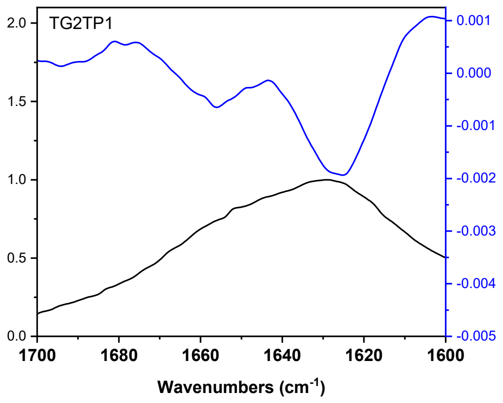

(c)

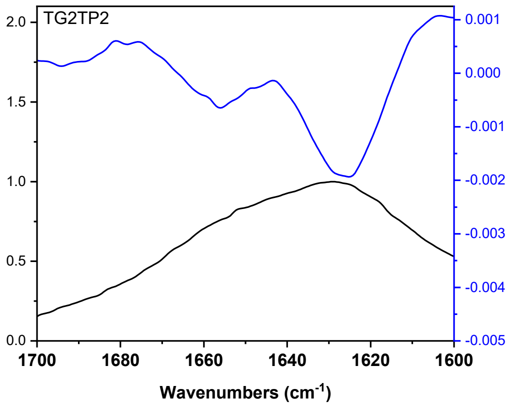

(d)

Figure S1. Second derivative of FTIR spectrum of (a) control; (b) TP2; (c) TG2TP1; and (d) TG2TP2 films.

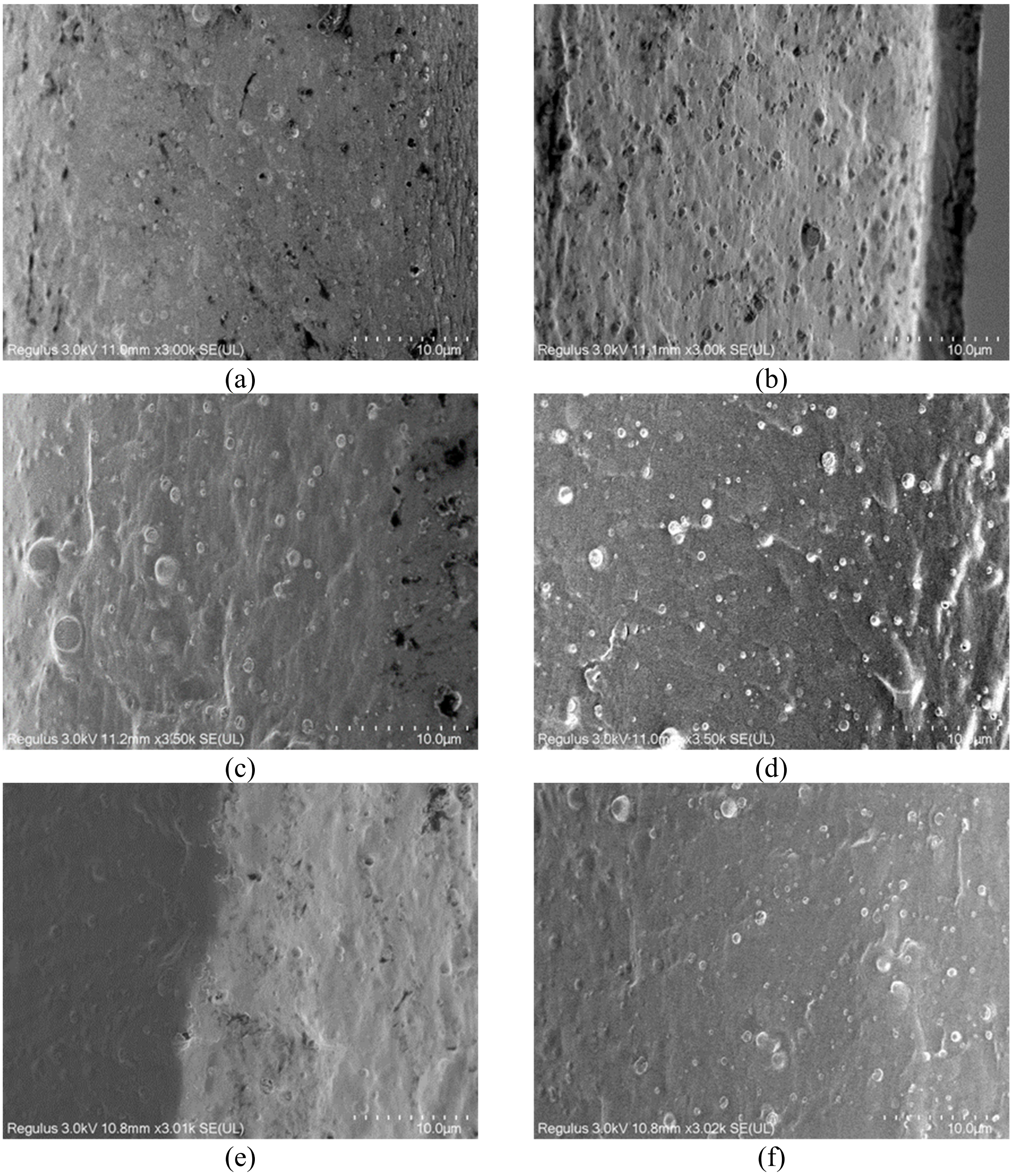

Figure S2. SEM images of cross-section of (a) control; (b) TP1; (c) TG1TP1; (d) TG2TP1; (e) TG1TP2; and (f) TG2TP2 films.
